# Supplementary material for: Phenotypic and genetic characteristics of retinal vascular parameters and their association with diseases
Source: Nat Commun. 2024 Nov 6;15:9593. doi: 10.1038/s41467-024-52334-1 (PMC11542103; doi:10.1038/s41467-024-52334-1)
Supplement: Supplementary file 3 — Reporting summary [file 41467_2024_52334_MOESM3_ESM.pdf]

nature portfolio

Corresponding author(s): Sven Bergmann, Sofía Ortín Vela, Michael Beyeler

Last updated by author(s): 19/07/2024

Reporting Summary

Nature Portfolio wishes to improve the reproducibility of the work that we publish. This form provides structure for consistency and transparency in reporting. For further information on Nature Portfolio policies, see our Editorial Policies and the Editorial Policy Checklist.

Statistics

For all statistical analyses, confirm that the following items are present in the figure legend, table legend, main text, or Methods section.

n/a	Confirmed	
☐	☒	The exact sample size (*n*) for each experimental group/condition, given as a discrete number and unit of measurement
☐	☒	A statement on whether measurements were taken from distinct samples or whether the same sample was measured repeatedly
☐	☒	The statistical test(s) used AND whether they are one- or two-sided<br>*Only common tests should be described solely by name; describe more complex techniques in the Methods section.*
☐	☒	A description of all covariates tested
☐	☒	A description of any assumptions or corrections, such as tests of normality and adjustment for multiple comparisons
☐	☒	A full description of the statistical parameters including central tendency (e.g. means) or other basic estimates (e.g. regression coefficient) AND variation (e.g. standard deviation) or associated estimates of uncertainty (e.g. confidence intervals)
☐	☒	For null hypothesis testing, the test statistic (e.g. F, t, r) with confidence intervals, effect sizes, degrees of freedom and P value noted<br>*Give P values as exact values whenever suitable.*
☐	☒	For Bayesian analysis, information on the choice of priors and Markov chain Monte Carlo settings
☐	☒	For hierarchical and complex designs, identification of the appropriate level for tests and full reporting of outcomes
☐	☒	Estimates of effect sizes (e.g. Cohen's *d*, Pearson's *r*), indicating how they were calculated

Our web collection on statistics for biologists contains articles on many of the points above.

Software and code

Policy information about availability of computer code

Data collection	UKBB, OphtalmoLaus, and Rotterdam Study data
Data analysis	Phenotypes extraction was developed, while exiting GWAS software was used

For manuscripts utilizing custom algorithms or software that are central to the research but not yet described in published literature, software must be made available to editors and reviewers. We strongly encourage code deposition in a community repository (e.g. GitHub). See the Nature Portfolio guidelines for submitting code & software for further information.

Data

Policy information about availability of data

All manuscripts must include a data availability statement. This statement should provide the following information, where applicable:

- Accession codes, unique identifiers, or web links for publicly available datasets
- A description of any restrictions on data availability
- For clinical datasets or third party data, please ensure that the statement adheres to our policy

The genetic and phenotypic UKBB data are available upon application to the UKBB, https://www.ukbiobank.ac.uk. Similarly for the replication data, RS and OphtalmoLaus, data can be made available upon request to researchers through a data transfer agreement. GWAS summary statistics will be available on the GWAS Catalog following publication.

Research involving human participants, their data, or biological material

Policy information about studies with human participants or human data. See also policy information about sex, gender (identity/presentation), and sexual orientation and race, ethnicity and racism.

Reporting on sex and gender	Findings apply to both sex.
Reporting on race, ethnicity, or other socially relevant groupings	Mostly Europeans
Population characteristics	age, sex, eye geometry, imaging device, and genomic princial componentes 1-10.
Recruitment	recruitment was done by the UKBB, Rotterdam Study and OphtalmoLaus
Ethics oversight	Access to the data to perform the study was approved by the UKBB, Rotterdam Study and OpthalmoLaus

Note that full information on the approval of the study protocol must also be provided in the manuscript.

Field-specific reporting

Please select the one below that is the best fit for your research. If you are not sure, read the appropriate sections before making your selection.

☒ Life sciences ☐ Behavioural & social sciences ☐ Ecological, evolutionary & environmental sciences

For a reference copy of the document with all sections, see nature.com/documents/nr-reporting-summary-flat.pdf

Life sciences study design

All studies must disclose on these points even when the disclosure is negative.

Sample size	71 494 subjects
Data exclusions	Subjects whose fundus images did not pass the quality control
Replication	Rotterdam Study (RS, N=8142<br>participants) and OphtalmoLaus (N=2 276 participants)
Randomization	Covariates were substracted
Blinding	encrypted eid

Reporting for specific materials, systems and methods

We require information from authors about some types of materials, experimental systems and methods used in many studies. Here, indicate whether each material, system or method listed is relevant to your study. If you are not sure if a list item applies to your research, read the appropriate section before selecting a response.

Materials & experimental systems

n/a	Involved in the study	
☒	☐	Antibodies
☒	☐	Eukaryotic cell lines
☒	☐	Palaeontology and archaeology
☒	☐	Animals and other organisms
☒	☐	Clinical data
☒	☐	Dual use research of concern
☒	☐	Plants

Methods

n/a	Involved in the study	
☒	☐	ChIP-seq
☒	☐	Flow cytometry
☒	☐	MRI-based neuroimaging

Plants

Seed stocks	no plants study
Novel plant genotypes	no plants study
Authentication	no plants study
